# Research progress on the treatment of perimenopausal insomnia with Chaihu Jia Longgu Muli decoction based on brain-intestine-bacteria axis: A review

**DOI:** 10.1097/MD.0000000000036537

**Published:** 2023-12-22

**Authors:** Yaolei Wang, Ruiqian Guan, Jifa Zhong, Qingchun Shi, Ziyu Ye, Limin Pan

**Affiliations:** a Heilongjiang University of Chinese Medicine, Harbin, China; b Second Affiliated Hospital of Heilongjiang University of Chinese Medicine, Harbin, China; c The First Affiliated Hospital of Heilongjiang University of Chinese Medicine, Harbin, China.

**Keywords:** bacteria axis, brain, Chaihu Jia Longgu Muli decoction, insomnia during perimenopausal period, intestine

## Abstract

With the progress and rapid societal development, women are confronted with multifaceted pressures in their lives, encompassing familial and other domains. Furthermore, during the perimenopausal phase, endocrine equilibrium is disrupted, leading to the emergence of psychological and physiological health challenges. Insomnia is a prevalent symptom among perimenopausal individuals. The brain-gut-bacteria axis assumes a pivotal role in the prevention, diagnosis, and management of perimenopausal insomnia. Chaihu Jia Longgu Muli decoction is a commonly prescribed remedy for addressing perimenopopausal insomnia. Consequently, this paper aims to investigate the interplay between the brain-gut-bacteria axis, intestinal microbiota, and the pathogenesis of perimenopausal insomnia. The study focuses on examining the regulatory effects of Chaihu Jia Longgu Muli decoction on the nervous system, intestinal microbiota, and the hypothalamus-pituitary-adrenal axis. Additionally, it explores the mechanisms underlying Hujia Longgu Muli decoction in mitigating perimenopausal insomnia.

## 1. Introduction

Perimenopause refers to the period starting from the disruption of ovarian function approaching menopause and extending up to 1 year after the last menstrual period.^[1]^ During this phase, the secretion of estrogen decreases due to ovarian function decline, leading to a series of physical and mental illnesses in women, collectively known as perimenopausal syndrome. Among these, the lack of sleep in terms of both duration and quality can result in impaired daytime social functioning, which is referred to as perimenopausal insomnia.^[2]^ According to relevant studies, approximately 1.5 million women transition from the normal physiological stage to the perimenopausal stage each year,^[3]^ with the proportion of women experiencing symptoms of perimenopausal insomnia reaching as high as 75% to 81%.^[4]^ Furthermore, research has demonstrated that adequate sleep is conducive to promoting metabolism, alleviating fatigue, delaying the aging process, and enhancing the body’s immune system.^[5]^ Prolonged insomnia disrupts the body’s ability to rest adequately, leading to reduced immune function, endocrine imbalances, and autonomic nervous system dysregulation, resulting in anxiety and depression.^[6]^

For the treatment of this condition, Western medicine’s sedatives or hormone therapy may have drawbacks such as dependency and an increased risk of breast and ovarian cancer in perimenopausal women. In contrast, traditional Chinese herbal formulations have a wealth of experience and demonstrated efficacy in treating perimenopausal insomnia, with Chaihu Jia Longgu Muli decoction being a commonly used and representative herbal prescription. Recent studies have shown that the gut microbiota has a certain degree of influence on brain activity, potentially linked to pathways involving the gut-brain axis, neurology, and endocrinology. Additionally, the gut microbiota plays a crucial role in the sleep-wake cycle, giving rise to the concept of the gut-brain-microbiota axis.^[7]^

This paper aims to explore the relationship between Chaihu Jia Longgu Muli decoction and perimenopausal insomnia from multiple perspectives, including gut microbiota, female hormones, the hypothalamus-pituitary-adrenal (HPA) axis, in order to delve into the treatment mechanisms of Chaihu Jia Longgu Muli decoction for perimenopausal sleep disturbances. This research further contributes to providing clinical support and theoretical foundations for the treatment of perimenopausal insomnia in traditional Chinese medicine.

## 2. Pathological studies on perimenopausal insomnia

The perimenopausal period is a significant phase for women, typically divided into 3 stages: early perimenopause, characterized by frequent irregular menstrual cycles; late perimenopause, marked by 1 year of amenorrhea; and early postmenopause, occurring within the first year after the last menstrual period.^[8–10]^ Typically, during late perimenopause and early postmenopause, the decline in ovarian function leads to a reduction in estrogen and progesterone levels, resulting in clinical manifestations primarily related to autonomic nervous system dysfunction, such as hot flashes, night sweats, insomnia, irritability, and others. Among these symptoms, insomnia is one of the most common,^[11]^ clinically referred to as perimenopausal insomnia.

The primary pathological changes in perimenopausal insomnia are observed during the rapid eye movement (REM) sleep stage. Research has shown that perimenopausal insomnia patients experience an extended latency period from wakefulness to REM, and there is a noticeable reduction in the total duration of REM sleep.^[12]^ Other studies^[13]^ have demonstrated a significant increase in the frequency of transitions from REM to wakefulness in perimenopausal insomnia patients, indicating that perimenopausal individuals are more likely to shift from REM to wakefulness.

Currently, it is believed that the substantial decrease in estrogen levels in perimenopausal insomnia patients is one of the significant factors contributing to the occurrence of REM sleep disturbances. Evidence suggests that women who undergo bilateral oophorectomy have an increased risk of REM sleep disorders.^[14]^ Estrogen therapy has a significant positive impact on the subjective sleep experience of perimenopausal individuals with sleep disorders, leading to improvements in sleep quality, extended total sleep duration, and reduced nighttime awakenings.^[15,16]^

## 3. Gut microbiota and perimenopausal insomnia

The total number of human cells is only about 1 to 10th of the total number of bacterial cells present in the human gut, which includes what is known as the “second genome,” the gut metagenome, with approximately 100 times more genes than the human genome. This metagenome cooperates with the human genome, making the gut microbiota an “invisible organ” within the human body and an essential component of the superorganism of the human body.^[17]^ Within the gut microbiota, the predominant bacterial phyla are Firmicutes and Bacteroidetes.^[18]^ The gut microbiota promotes intestinal mucosal integrity, provides essential nutrients to the host, defends against pathogens, and maintains homeostasis while interacting with the environment and the host. It also plays a crucial role in cognition and behavior.^[19]^

Under normal conditions, around 60% of circulating estrogens are bound in the form of glucuronic acid and excreted into bile, with subsequent reabsorption in the epithelial cells of the intestinal mucosa. The gut microbiota can produce β-glucuronidase, which unbinds estrogens and glucuronic acid, increasing the levels of estrogens in the body. However, around the age of 50, as ovarian function declines in women, estrogen production decreases, giving rise to a series of syndromes known as perimenopausal syndrome, with insomnia being a primary symptom. Research has shown that the structure of the gut microbiota changes significantly in perimenopausal women, with a notable decrease in Bifidobacteria and an increase in bacteria from the Enterobacteriaceae family, Streptococcus, and Enterococcus.^[20]^ In a study of postmenopausal women, estrogen metabolite levels in urine were positively correlated with the proportion of Firmicutes and the diversity of the gut microbiota.^[21]^ The gut microbiota is involved in the metabolism and reabsorption of estrogens, thereby affecting the estrogen levels in the bodies of perimenopausal women. Estrogen is not only associated with female reproductive functions but also plays a role in the central nervous system and the circadian rhythms of women.^[22]^ Research indicates that rats that have had their ovaries removed experience an increase in both REM and non-REM sleep, leading to circadian rhythm disruption. With estrogen supplementation, the circadian rhythm can be restored to normal under the influence of estrogen.^[23]^

Furthermore, gut microbiota, such as Bifidobacteria, can produce various neurotransmitters through their metabolism or influence on intestinal secretory cells, including 5-Hydroxytryptamine (5-HT), cortisol (CORT), dopamine (DA), and more. These neurotransmitters can be absorbed into the bloodstream through intestinal epithelial cells and affect the central nervous system and the HPA axis. Changes in the activity and levels of neurotransmitters in the hypothalamus are associated with H-P-A axis dysfunction, further contributing to the development of insomnia.^[24]^ The gut microbiota can also regulate central neurotransmitters by altering their precursor levels. For example, research has shown that infant Bifidobacteria can increase plasma tryptophan levels, thus affecting the transmission of central serotonin 5-HT. Additionally, the gut microbiota can synthesize and release neurotransmitters directly, such as Lactobacillus and Bifidobacteria producing γ-aminobutyric acid, and Escherichia, Bacillus, and yeast synthesizing epinephrine, among others.

In addition, melatonin is another important mechanism leading to reduced nighttime sleep quality in perimenopausal women. Melatonin is a high-ranking endogenous time-keeping factor that plays a crucial role in regulating the sleep-wake cycle.^[25]^ Research has shown that melatonin secretion is age-dependent, with levels decreasing as individuals age, which can lead to sleep disturbances.^[26]^ Melatonin and its receptors are widely distributed in the gastrointestinal tract, with the gut being the second most significant source of melatonin after the pineal gland.^[27]^ Melatonin in the gut is mainly produced by endocrine cells in the intestine and the gut microbiota.^[28]^ In conclusion, the relationship between gut microbiota and perimenopausal insomnia is closely intertwined.

## 4. Chaihu Jia Longgu Muli decoction and perimenopausal insomnia

The 107th verse of the “Shanghan Lun” states: “In the 8th or 9th day of Shanghan (febrile disease), if there is chest fullness, difficulty urinating, delirium, overwhelming sensation in the whole body, and inability to turn to either side, Chaihu Jia Longgu Muli decoction is the primary treatment”. This verse implies that on the 8th or 9th day after the onset of a febrile disease, when a pattern resembling Chaihu (Bupleurum) decoction is present, the pathogenic factors have invaded the Shaoyang (lesser yang) stage. However, due to the lack of thorough examination, improper purgation therapy may damage the vital Qi, leading to symptoms such as chest discomfort, persistent uneasiness, which signifies that the Chaihu pattern has not been resolved. The internal heat rises, causing restlessness and a sense of anxiety. Urination is obstructed due to the impairment of water metabolism. If heat accumulates in the body, it can result in confusion and delirium. If dampness stagnates in the body, individuals may experience a heavy feeling and be unable to easily change their position. In such cases, Chaihu Jia Longgu Muli decoction is recommended for treatment.^[29]^

This prescription is composed of twelve ingredients, including Chaihu (Bupleurum), Guizhi (Cinnamomum cassia), Fuling (Poria cocos), Longgu (Fossilia ossis mastodi), Muli (Ostrea gigas), Qiandang (Cinnabaris), and Chuanjiao (Zanthoxylum bungeanum).^[30]^ Chaihu in the formula releases liver constraint while also dispelling Shaoyang (lesser yang) pathogenic heat. Banxia (Pinellia ternata) dries dampness and transforms phlegm. Dahuang (Rheum palmatum) purges stagnation and unblocks channels while also clearing heat and restlessness. Longgu and Muli, when used together, strongly calm the spirit and nourish Yin, while Renshen (Panax ginseng) supplements Qi and calms the spirit. Guizhi warms and facilitates Qi movement to disperse stagnation. Fuling invigorates the spleen and eliminates dampness. Huangqin (Scutellaria baicalensis) clears heat and dries dampness. Cinnabaris has astringent and sedative properties, while Dazao (Ziziphus jujuba) nourishes Qi and blood. Shengjiang (Zingiber officinale) disperses cold and releases the exterior.

Traditional Chinese medicine theory suggests that the main pathological mechanisms of perimenopausal insomnia include Qi and blood disharmony, organ imbalance, and Yin-Yang disharmony.^[31]^ In the “Lingshu: Discussion on the Nourishment of the Vital and Defensive Qi”, it is stated: “In the elderly, their Qi and blood are deficient. Their nourishing Qi is reduced, and the defensive Qi invades inward, resulting in lack of concentration during the day and inability to sleep at night.” This indicates that due to the weakness of nourishing Qi, individuals cannot concentrate during the day and have difficulty falling asleep at night, which closely resembles the clinical presentation of perimenopausal sleep disorders.^[32]^ Prolonged insomnia leads to exhaustion of nourishing and blood, preventing the control of Yang, reducing organ function, inhibiting the functions of the Shaoyang “pivot”, and causing prolonged disturbance, eventually giving rise to internal heat. At the same time, the lack of Yin leads to an imbalance with excessive Yang, making it difficult to sleep at night. Chaihu Jia Longgu Muli decoction can disperse Shaoyang pathogenic heat, promote the smooth flow of Qi, and soothe emotional stress, while also strongly calming the spirit, making it suitable for the treatment of perimenopausal insomnia.

Pharmacological studies have shown that Chaihu Jia Longgu Muli decoction has an inhibitory effect on MEK/ERK phosphorylation, reducing oxidative stress damage in individuals and improving the symptoms of insomnia.^[33]^ Clinical trials have demonstrated that Chaihu Jia Longgu Muli decoction significantly improves the sleep quality of insomnia patients. After 2 weeks of treatment, clinical results become evident, and even after stopping treatment for some time, sleep quality remains at a high level.^[34]^ With a deeper understanding of the classical formula and adjustments to the prescription, Chaihu Jia Longgu Muli Decoction’s scope of treatment has expanded to include conditions such as depression, coronary heart disease, primary hypertension, and more. The brain-gut-microbiota axis plays a crucial role in the treatment of perimenopausal insomnia, and studies have indicated that Chaihu Jia Longgu Muli decoction significantly impacts the gut microbiota.^[35]^ In recent years, the brain-gut-microbiota axis has played a crucial role in the treatment of perimenopausal insomnia, and Chaihu Jia Longgu Muli decoction’s dual effects on the gut system and microbiota, as well as the nervous system, provide a basis for understanding its mechanisms in the treatment of perimenopausal insomnia.

## 5. The relationship between perimenopausal insomnia and the brain-gut axis

Numerous studies have demonstrated that the diversity of gut microbiota in insomnia patients is often higher, and it exhibits a significant correlation with different subtypes of insomnia.^[36]^ Although the pathogenesis of perimenopausal insomnia is complex and not yet fully understood, the prevailing mechanism, accepted in the academic community, is the result of a combination of factors, including the decline in female reproductive function and the associated reduction in estrogen levels, which triggers a cascade of neuroendocrine events involving the hypothalamic-pituitary-gonadal axis.^[37]^ Research has affirmed that various pathways, encompassing immune, neural, and endocrine interactions, establish communication between the gut and the brain, exerting bidirectional effects on sleep quality and psychological well-being.^[38]^

Furthermore, an exploration of the fundamental pathological mechanisms of perimenopausal insomnia suggests that imbalances in the Heart-Kidney relationship, as described in traditional Chinese medicine, are involved. Specifically, an analysis of perimenopausal insomnia patients and non-insomnia control groups revealed differences in the composition of the gut microbiota, including the presence of Roseburia, Lactobacillus, and Prevotella, among others. After treatment with Tianwang Buxindan, a herbal remedy for perimenopausal insomnia, there were substantial changes in the abundance and proportions of gut microbiota components among the patients.^[39]^

In conclusion, the brain-gut axis appears to be a crucial contributing factor to the occurrence and development of perimenopausal insomnia. The relationship between the brain and the gut, as perceived in traditional Chinese medicine, is discernible in texts such as the “Huangdi Neijing” (Yellow Emperor’s Inner Canon) and the “Lingshu: Yingwei Shenghui”. These texts highlight the decline of vital qi and blood during the perimenopausal stage, which results in diminished mental clarity during the day and difficulty falling asleep at night. Insights from traditional Chinese medicine, particularly the concept of the brain-gut connection, provide valuable physiological and pathological evidence that underscores the intricate relationship between these 2 systems. Additionally, the theory presented in the “Suwen: Neitiao Lun” elaborates on the impact of disrupted gastric function on sleep, further reinforcing the profound influence of the gut on sleep patterns. The approach of addressing sleep disturbances through the regulation of the spleen and stomach has matured over time and is well-documented in texts such as Zhang Yuqing “Yian” from the late Qing Dynasty.^[40]^ In summary, the brain-gut axis offers an essential perspective on the interplay between the gastrointestinal system and the central nervous system, enhancing the scientific underpinning of traditional Chinese medical theory. The aforementioned Chinese medical concepts provide substantial physiological and pathological evidence supporting the connection between the brain and the gut.

## 6. Regulatory effects of Chaihu Jia Longgu Muli decoction on the nervous system

During the perimenopausal phase, the levels of estrogen in the internal environment are adversely affected due to declining ovarian function. Simultaneously, reduced sensitivity of estrogen receptor β leads to significant interference with brain-derived neurotrophic factor and disrupts its expression and signaling. This, in turn, affects downstream pathways and the expression of 5-Hydroxytryptamine 2A receptors. Consequently, perimenopausal women have an increased likelihood of experiencing sleep disturbances.^[41]^

Research has indicated that low doses of Chaihu Jia Longgu Muli decoction can reduce serotonin levels in the rat brain. In rats with perimenopausal insomnia, this decoction plays a role in regulating sleep, promoting wakefulness, and also impacting non-rapid eye movement sleep to some extent.^[42]^ Clinical trials conducted by Liu Jing, Jin Yabei, and others demonstrated that when combined with auricular acupuncture, Chaihu Jia Longgu Muli decoction with modifications led to lower PSQI scores compared to the control group receiving estradiol.^[43]^ Additionally, it increased the levels of 5-HT and DA compared to the control group, suggesting that this combination treatment can enhance sleep quality in patients by regulating 5-HT and DA levels.

Furthermore, Chaihu Jia Longgu Muli decoction can modulate serotoninergic neurons in the median raphe nucleus, preventing the occurrence of insomnia.^[44]^ It significantly decreases plasma adrenocorticotropic hormone and corticosterone levels,^[45]^ thereby inhibiting the excitability of the HPA axis.^[46]^ This effect helps prevent sleep disturbances and mood disorders in perimenopausal women.

Experimental evidence reveals that the active ingredient CHJLMD70E^[47]^ in Chaihu Jia Longgu Muli decoction mediates the ERβ/brain-derived neurotrophic factor/TrkB/5-HT2A signaling pathway.^[48]^ This regulation contributes to balancing the hypothalamic-pituitary-ovarian axis, improving the sleep-wake cycle mechanism, and significantly increasing sleep duration in affected individuals.^[49]^

In summary, Chaihu Jia Longgu Muli decoction exhibits regulatory effects on the nervous system, specifically in terms of neurotransmitters, neurotrophic factors, and hormonal pathways. This herbal remedy has shown promise in mitigating the sleep disturbances often experienced by perimenopausal women, offering a potential solution to the challenges associated with this transitional phase.

## 7. Chaihu Jia Longgu Muli decoction and the gut microbiota

The human gut microbiota, evolved through millennia of coexistence with the human host, maintains a complex and intricate relationship with the body. The gut microbiota constitutes a vital component of the human ecosystem, significantly influencing various physiological functions and bearing recognized value in the prognosis and treatment of global diseases. Currently, a high degree of correlation has been observed between perimenopausal insomnia and the gut microbiota. Therefore, investigating the regulatory effects of Chaihu Jia Longgu Muli decoction on the gut microbiota allows us to further understand its role in the context of the brain-gut-microbiota axis concerning perimenopausal insomnia.

Wang Jiayun et al^[50]^ employed the “Five Principles” of Traditional Chinese Medicine Quality Marker prediction and analysis to identify key bioactive compounds in Chaihu Jia Longgu Muli decoction that contribute to its anti-insomnia effects. The primary components responsible for these effects were identified as saikosaponins, Poria cocos polysaccharides, baicalin, and cinnamaldehyde. Sun Bing et al^[51]^ discovered that saikosaponins can counteract para-chlorophenylalanine, leading to increased serotonin levels and promoting sleep. Tang C et al^[52]^ co-cultivated Chaihu extract with human fecal suspensions, revealing deglycosylation and dehydration reactions as the primary metabolic pathways for saikosaponins. Furthermore, other constituents of Chaihu were similarly metabolized by the gut microbiota, underscoring the potential role of gut microbiota metabolism as a significant pathway for the pharmacological action of saikosaponins.

Sun Jingliang et al^[53]^ summarized that the gut microbiota has the capacity to influence the nervous system, resulting in conditions such as depression and anxiety. Chaihu Jia Longgu Muli decoction exhibited gut microbiota-regulating effects in rats with schizophrenia, which, in turn, ameliorated depressive and anxious states. This demonstrates the bidirectional regulatory effects of Chaihu Jia Longgu Muli decoction on the gut microbiota and the nervous system.

Currently, no research institution has conducted a comprehensive analysis of the gut microbiota metagenome related to Chaihu Jia Longgu Muli decoction’s treatment of sleep disturbances in perimenopausal women. Therefore, the specific impacts of this decoction on the human gut microbiota remain inconclusive. However, the studies summarized above indicate a strong correlation between the gut microbiota, Chaihu Jia Longgu Muli decoction absorption and utilization, and the treatment of perimenopausal insomnia. Further research in this area is warranted.

## 8. Regulation of the HPA axis function by Chaihu Jia Longgu Muli decoction

The HPA axis constitutes a neuroendocrine system responsible for regulating the body’s stress response. Activation of the HPA axis, characterized by increased secretion of corticotropin-releasing hormone (CRH), leads to elevated concentrations of adrenocorticotropic hormone and CORT. Overactivity of the HPA axis results in arousal effects.^[54]^ Therefore, long-term sleep disturbances involve the dysregulation of the HPA axis as one of the contributing factors in neurobiology.^[55]^

Wang Xiaobin, Xu Rui, and others^[56]^ conducted a study to investigate the mechanism of Chaihu Jia Longgu Muli decoction by measuring the levels of CRH and CORT in rats before and after administration using an enzyme-linked immunosorbent assay. The experimental results indicated that in rats with depressive-like symptoms, the levels of CRH and CORT decreased after taking Chaihu Jia Longgu Muli decoction. This outcome supports the notion that the decoction inhibits HPA axis overactivity through a certain pathway.

Chen Yali, Hou Jiqiu, and colleagues^[57]^ also examined the impact of Chaihu Jia Longgu Muli decoction on the HPA axis in rats with anxiety and concomitant myocardial infarction. Their research found that the decoction improved heart function in rats with anxiety and myocardial infarction and effectively regulated HPA axis function, restoring it to a more normal state. However, given the multifactorial influences and regulatory elements affecting the HPA axis, it remains unclear whether Chaihu Jia Longgu Muli decoction indirectly or directly controls HPA axis function. Further research is needed to elucidate this.

Therefore, in the treatment of perimenopausal insomnia, the regulatory effect of Chaihu Jia Longgu Muli decoction on the HPA axis function in women is of paramount importance.

## 9. Discussion

In summary, the perimenopausal period is of paramount importance for women. During this phase, ovarian function begins to decline, and sex hormone levels undergo significant fluctuations. Traditional medicine views this stage as a period of “Yin-Yang imbalance” within the body, resulting in a range of physical and psychological symptoms. Insomnia, as one of the most common ailments among perimenopausal women, presents unique challenges due to the influence of specific physiological changes during this stage and is considered a clinical conundrum in both Western and traditional gynecology.^[58]^

**Figure 1.**
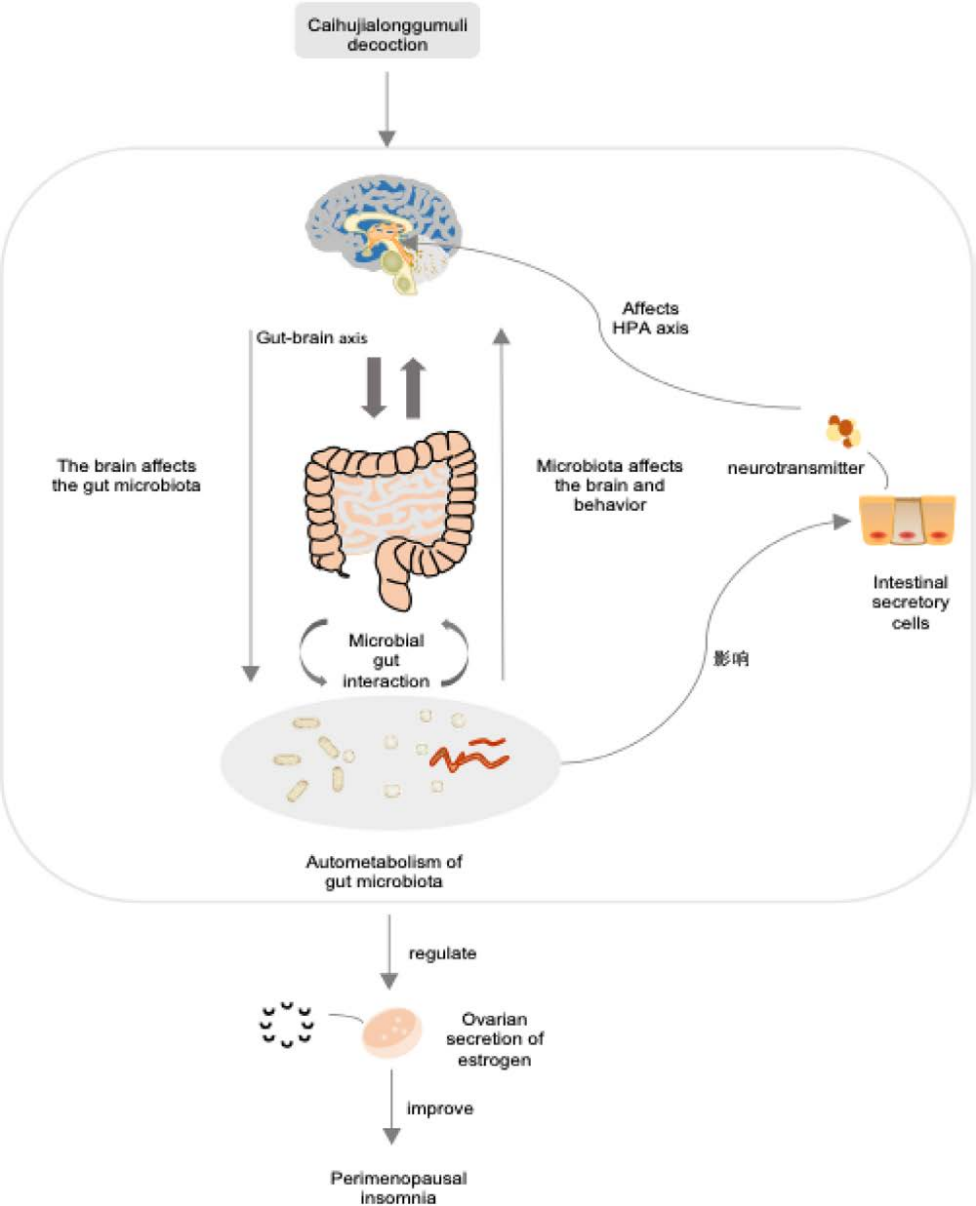
Mechanism of the treatment of perimenopausal insomnia with Chaihu Jia Longgu Muli decoction based on brain-intestine-bacteria axis.

Moreover, the gut microbiota constitutes an essential component of the human ecological system, playing a crucial role in regulating the body’s functions. In the period before and after menopause, as reproductive functions decline, the body’s preexisting equilibrium is disrupted, and a new balance is yet to be fully established. During this phase, the gut microbiota, through its own activities and metabolic byproducts, alters the secretion levels of estrogen and melatonin in the body, thereby affecting neurotransmitter levels and activity. It exerts a bidirectional influence on the gut-brain axis, becoming one of the key mechanisms underlying the occurrence of perimenopausal insomnia. (Fig. 1)

Conventional treatment often involves the use of Western medicine, with patients receiving sedative-hypnotic drugs or hormone replacement therapy. However, prolonged use of Western medication may lead to side effects, drug dependence, recurrent symptoms, and, in some cases, a higher risk of uterine and breast cancer.^[59,60]^ On the other hand, the components of Chaihu Jia Longgu Muli decoction, through their regulation of different pathways, such as the HPA axis, gut microbiota, estrogen, melatonin, and others, promote the restoration of homeostasis within the body. They work together to bring the body back to the state referred to in traditional Chinese medicine as “Yin-Ping-Zi-He” (Harmonious Yin and Yang). This collaborative approach demonstrates its efficacy in treating perimenopausal insomnia in women.

Research has shown that the combination of Western medicine and Chaihu Jia Longgu Muli decoction has a synergistic effect, reduces adverse reactions, and enjoys high patient compliance. However, there remains a lack of research regarding the effective components of traditional Chinese medicine for the treatment of perimenopausal insomnia, and human trials are scarce. While discussions on the mechanisms of the gut-brain axis are abundant, their application in clinical practice remains limited. Therefore, it is essential to allocate more resources to research on the brain-gut-microbiota axis, fully explore the preventive and therapeutic roles of the gut microbiota in human diseases, and integrate these findings into clinical practice. This approach can further advance the scientific understanding of traditional medical theories and provide a new clinical perspective for the treatment of perimenopausal insomnia.

## Author contributions

**Formal analysis:** Qingchun Shi.

**Investigation:** Ruiqian Guan.

**Supervision:** Jifa Zhong, Ziyu Ye, Limin Pan.

**Writing – original draft:** Yaolei Wang.

**Writing – review & editing:** Yaolei Wang.

## References

[1] Harlow SD Gass M Hall JE. Executive summary of the stages of reproductive aging workshop+10: addressing the unfinished agenda of staging reproductive aging. Fertil Steril. 2012;97:843–51. 22341880 10.1016/j.fertnstert.2012.01.128 PMC3340904

[2] Uhlig BL Hagen K Engstrøm M. The relationship between obstructive sleep apneaand insomnia: a population-based cross-sectional polysomnographic study. Sleep Med. 2018;17:126–33. 10.1016/j.sleep.2018.10.026 30554056

[3] Nowakowski S Meliska CJ Martinez LF. Sleep and menopause. Curr Neurol Neurosci Rep. 2009;9:165–72. 19268040 10.1007/s11910-009-0025-6

[4] David AK James LA Todd A. Insomnia symptoms and short sleep predict anxiety and worry in response to stress exposure: a prospective cohort study of medical interns. Sleep Med. 2019;55:40. 30763868 10.1016/j.sleep.2018.12.001 PMC7045299

[5] Ma K Chen Y Dong M. Clinical study on treatment of perimenopausal sleep disorder of kidney deficiency and blood stasis syndrome by tonifying kidney, activating blood circulation and tranquilizing mind. Chin J Tradit Chin Med. 2019;44:1069–74. 10.19540/j.cnki.cjcmm.20181225.002 30989965

[6] Jiang Y Liu Y Li Y. Effect of pestle needle combined with five-tone therapy on anxiety and depression of insomnia patients with digestive system malignant tumor of heart and spleen deficiency type. Nurs Res. 2018;32:2723–6.

[7] Bathgate CJ Edinger JD Krystal AD. Insomnia patients with objective short sleep duration have a blunted response to cognitive behavioral therapy for insomnia. Sleep. 2017;40:zsw012. 28364452 10.1093/sleep/zsw012 PMC6084751

[8] Soules MR Sherman S Parrott E. Executive summary: stages of reproductive aging workshop (STRAW). Climacteric. 2001;4:267–72. 11770182

[9] Monteleone P Mascagni G Giannini A. Symptoms of menopause-global prevalence, physiology and implications. Nat Rev Endocrinol. 2018;14:199–215. 29393299 10.1038/nrendo.2017.180

[10] Xu X Zhang H. Mediating effect of anxiety on the relationship between sleep disorders and quality of life in perimenopausal women. Chin J Behav Med Brain Sci. 2019;28:592–596.

[11] Zou J Xie X Li W. Clinical observation on acupuncture combined with refined moxibustion for treatment of perimenopausal insomnia of heart-kidney disharmony type. J Guangzhou Univ Tradit Chin Med. 2023;40:1982–8.

[12] Terashima K Mikami A Tachibana N. Sleep characteristics of menopausal insomnia: a polysomnographic study. Psychiatry Clin Neurosci. 2004;58:179–85. 15009824 10.1111/j.1440-1819.2003.01214.x

[13] Zhao Y Chen Q Jiang C. Clinical characteristics of insomnia in perimenopausal women by polysomnography. J Third Mil Med Univ. 2021;43:1854–9.

[14] Gallicchio L Whiteman MK Tomic D. Type of menopause, patterns of hormone therapy use, and hot flashes. Fertil Steril. 2006;85:1432–40. 16566933 10.1016/j.fertnstert.2005.10.033

[15] Savolainen-Peltonen H Hautamäki H Tuomikoski P. Health-related quality of life in women with or without hot flashes: a randomized placebo- controlled trial with hormonetherapy. Menopause. 2014;21:732–9. 24219882 10.1097/GME.0000000000000120

[16] Welton AJ Vickers MR Kim J. Health related quality of life after combined hormone replacement therapy: randomised controlled trial. BMJ. 2008;337:a1190. 18719013 10.1136/bmj.a1190 PMC2518695

[17] Dinan TG Stilling RM Stanton C. Collective uncon-scious: how gut microbes shape human behavior. J Psychiatr Res. 2015;63:1–9. 25772005 10.1016/j.jpsychires.2015.02.021

[18] Rinninella E Raoul P Cintoni M. What is the healthy gut microbiota composition? A changing ecosystem across age, environment, diet, and diseases. Microorganisms. 2019;7:14. 30634578 10.3390/microorganisms7010014 PMC6351938

[19] Adak A Khan MR. An insight into gut microbiota and its functionalities. Cell Mol Life Sci. 2019;76:473–93. 30317530 10.1007/s00018-018-2943-4 PMC11105460

[20] Guo Z. Study on intestinal microbial community structure in patients with climacteric syndrome. Chin J Microecol. 2015;27:477–9.

[21] Fuhrman BJ Feigelson HS Flores R. Associations of the fecalmicrobiome with urinary estrogens and estrogen metabolites in postmenopausal women. J Clin Endocrinol Metab. 2014;99:4632–40. 25211668 10.1210/jc.2014-2222 PMC4255131

[22] Joffe H Petrillo LF Koukopoulos A. Increased estradiol and improved sleep, but not hotflashes, predict enhanced mood during the menopausal transition. J Clin Endocrinol Metab. 2011;96:1044–54. 10.1210/jc.2010-2503 PMC3135198 21525161

[23] Deurveilher S Seary ME Semba K. Ovarian hormones promote recovery from sleep deprivation by increasing sleep intensity in middle-aged ovariectomized rats. Horm Behav. 2013;63:566–76. 23454003 10.1016/j.yhbeh.2013.02.011

[24] Greer SM Goldstein AN Knutson B. A genetic polymorphism of the human dopamine transporter determines the impact of sleep deprivation on brain responses to rewards and punishments. J Cogn Neurosci. 2016;28:803–10. 26918589 10.1162/jocn_a_00939

[25] Morgan D Tsai SC. Sleep and the endocrine system. Crit Care Clin. 2016;31:403–18. 10.1016/j.ccc.2015.03.004 26118912

[26] Zeng Q Xiong Z. Etiology of perimenopausal sleep disorders. Chin Pract Gynecol Obstetr. 2018;34:821–3.

[27] Tan B Ren W Yin Y. Secretion of melatonin in gastrointestinal tract and its physiological function. J Anim Nutr. 2018;30:1207–16.

[28] Yano JM Yu K Donaldson GP. Indigenous bacteria from the gut microbiota regulate host serotonin biosynthesis. Cell. 2015;161:264–76. 25860609 10.1016/j.cell.2015.02.047 PMC4393509

[29] Zhu M Feng X Hao W. Examples of Feng Shilun’s application of Chaihujiagulongmu decoction. Chin J Tradit Chin Med. 2022;37:6518–21.

[30] He Z Xiong Y. Chief Physician Xiong Yan: examples of proved cases of Chaihujialongguoyushi decoction. Guangming Tradit Chin Med. 2021;36:2703–6.

[31] Jing Q Ren L. Discussion on TCM pathogenesis of perimenopausal insomnia from kidney-yang deficiency. Liaoning J Tradit Chin Med. 2020;47:71–3.

[32] Wang M Wang Y Huang N. Syndrome differentiation and treatment of perimenopausal depression and insomnia from the perspective of “no sperm in the daytime and no sleep at night”. Chin J Tradit Chin Med. 2018;33:4261–3.

[33] Jin X Zhang W Zhang D. To explore the mechanism of Chaihu plus Longgu Muli decoction in the treatment of insomnia rats based on MEK/ERK pathway. Pharmacol Clin Tradit Chin Med. 2020;36:51–4.

[34] Wang B Zhang T Qiu L. Clinical study on the treatment of insomnia with Chaihu Plus Longgu Muli decoction based on Zhongjing’s Chaihu prescription and syndrome theory. Chin J Tradit Chin Med. 2016;34:1430–3.

[35] Li Z Pang M Lin P. Effect of Chaihu Longgu Muli decoction on intestinal microflora diversity in schizophrenic rats based on 16s rRNA technology. Chin J Exp Prescr. 2019;25:1–8.

[36] Luo J Wu Y Huang F. Study on the difference of intestinal flora in patients with primary insomnia of different TCM syndromes. Chin J Inf Tradit Chin Med. 2018;25:28–34.

[37] Du C Zhao Y Liu H. Discussion on the essential connotation of heart-kidney disharmony syndrome in perimenopausal insomnia based on the axis of “intestinal flora-intestine-brain”. Chin J Tradit Chin Med. 2021;36:6975–8.

[38] Li Y Hao Y Fan F. The role of microbiome in insomnia, circadian disturbance and depression. Front Psychiatry. 2018;9:669. 30568608 10.3389/fpsyt.2018.00669 PMC6290721

[39] Yang X Xiao H Zeng Y. Tianwang Buxin granules influence the intestinal flora in perimenopausal insomnia. Biomed Res Int. 2021;2021:9979511. 34825005 10.1155/2021/9979511 PMC8610686

[40] Ji J Wang J. The research and application of “stomach disharmony leads to restlessness” by physicians in the past dynasties. China Mod Distance Educ Tradit Chin Med. 2020;18:43–7.

[41] Chhibber A Woody SK Rumi MK. Estrogen receptor β de- ficiency impairs BDNF– 5-HT2A signaling in the hippocampus of female brain: a possible mechanism for menopausal depression abstract. Psychoneuroendocrinology. 2017;82:107–16. 28544903 10.1016/j.psyneuen.2017.05.016 PMC5523821

[42] Wang W Sun F Li F. Effect of Chaihu plus Longgu Muli decoction on monoamine transmitters in anxiety model rats. New Drugs Clin Pharmacol Tradit Chin Med. 2008;(05):340–2.

[43] Liu J Jin Y. Curative effect of modified Chaihu Longgu Muli decoction combined with auricular acupoint program on perimenopausal patients with sleep disorders [J/OL]. Chin J Tradit Chin Med. 2023:1–6[2023-12-14]. Available at: http://kns.cnki.net/kcms/detail/21.1546.R.20221019.1019.004.html.

[44] Zhao Z. Clinical Sleep Disorders. Shanghai: Second Military Medical University Press; 2003.

[45] Kang D Qu R Zhu W. Effect of Chaihu plus Longgu Muli decoction on hypothalamic-pituitary-adrenal axis in depressed animals. Chin J Clin Pharmacol Ther. 2005;11:1231–5.

[46] Zhao L. Study on the Internal Correlation of Hyperplasia of Mammary Glands Treated with Liver and Kidney Simultaneously and HPA and HPO Axis. Beijing: Beijing University of Traditional Chinese Medicine; 2014.

[47] Dunana Yu S Huang L. Study on the effective parts of Chaihu plus Longgu Muli Decoction for improving sleep in perimenopausal female mice. New Chin Med Clin Pharmacol. 2014;25:556–9.

[48] Yan J. Effects of Chaihu plus Longgu Muli Decoction on anxiety-like and depression-like behaviors in perimenopausal sleep deprived mice. Harbin: Heilongjiang University of Chinese Medicine; 2020.

[49] Zhang K Zhang B Zhu S. Research progress of Chaihu plus Longgu Muli decoction in the treatment of insomnia. Chin J Drug Depend. 2021;30:161–6.

[50] Wang J Gao Z Gao L. Research progress and quality marker prediction analysis of classic prescription Chaihujiagulongmu decoction. [J/OL]. Chin J Tradit Chin Med. 2023;41:29–36. Available at: http://kns.cnki.net/kcms/detail/21.1546.R.20221205.1641.019.html.

[51] Sun B Hao H Zheng K. A preliminary study on the mechanism of saikosaponin regulating sleep rhythm in cats. Tianjin Med Univ J. 2000:274–6.

[52] Tang C Fu Q Chen X. The biotransformation of Bupleuri Radix by human gut microbiota. Xenobiotica. 2020;50:1011–22. 31858877 10.1080/00498254.2019.1707908

[53] Sun J Xu T. Based on the theory of brain-gut axis, Chaihu plus Longgu Muli decoction was used to treat metabolic syndrome with the method of “treating both mind and body”. Hebei J Tradit Chin Med. 2021;43:1025–8.

[54] Dressle RJ Feige B Spiegelhalder K. HPA axis activity in patients with chronic insomnia: a systematic review and meta-analysis of case-control studies. Sleep Med Rev. 2022;62:101588. 35091194 10.1016/j.smrv.2022.101588

[55] Vargas I Vgontzas AN Abelson JL. Altered ultradian cortisol rhythmicityas a potential neurobiologic substrate for chronic insomnia. Sleep Med Rev. 2018;41:234–43. 29678398 10.1016/j.smrv.2018.03.003 PMC6524148

[56] Wang X Xu R Kong M. Effects of Chaihu plus Longgu Muli decoction on forced swimming behavior and HPA axis in chronic stress depressive rats. J Harbin Med Univ. 2014;48:198–201.

[57] Chen Y Hou J Wang W. Effect of Chaihu plus Longgu Muli decoction on HPA axis in rats with myocardial infarction and anxiety. J Cardio Cerebrovasc Dis Integr Tradit Chin West Med. 2022;20:2747–51 + 2756.

[58] Li RX Ma M Xiao XR. Perimenopausal syndrome and mood disorders in perimenopause: prevalence, severity, relationships, and risk factors. Medicine (Baltimore). 2016;95:e4466. 27512863 10.1097/MD.0000000000004466 PMC4985318

[59] Han L Yang Q Su X. Effect of auricular point sticking combined with psychological intervention on hormone replacement therapy for female perimenopausal syndrome. J Changchun Univ Tradit Chin Med. 2017;33:461–3.

[60] Tang J Liu Q Ma Y. Clinical study on 30 cases of perimenopausal syndrome treated with integrated traditional Chinese and western medicine. Jiangsu J Tradit Chin Med. 2019;51:45–7.

